# PD-1/PD-L1 Targeting in Breast Cancer: The First Clinical Evidences are Emerging—A Literature Review

**DOI:** 10.3390/cancers11071033

**Published:** 2019-07-22

**Authors:** Gabrielle Planes-Laine, Philippe Rochigneux, François Bertucci, Anne-Sophie Chrétien, Patrice Viens, Renaud Sabatier, Anthony Gonçalves

**Affiliations:** 1 Department of Medical Oncology, Aix-Marseille University, Inserm U1068, CNRS UMR7258, Institute Paoli-Calmettes, 13009 Marseille, France; 2 CRCM—Tumor Immunology Laboratory, Aix-Marseille University, Inserm U1068, CNRS UMR7258, Institute Paoli-Calmettes, 13009 Marseille, France; 3 CRCM—Predictive Oncology Laboratory, Aix-Marseille University, Inserm U1068, CNRS UMR7258, Institute Paoli-Calmettes, 13009 Marseille, France

**Keywords:** breast cancer, immunotherapy, PD1, PDL1, triple-negative breast cancer

## Abstract

Recently, the development of immunotherapy through the immune checkpoint blockade led to long-lasting responses in several types of cancers that are refractory to conventional treatments, such as melanoma or non-small cell lung cancer. Immunotherapy has also demonstrated significant improvements in various other types of cancers. However, breast cancer remains one of the tumors that have not experienced the explosion of immunotherapy yet. Indeed, breast cancer was traditionally considered as being weakly immunogenic with a lower mutational load compared to other tumor types. In the last few years, anti-PD1/PD-L1 (Programmed death-ligand 1) agents have been evaluated in breast cancer, particularly in the triple negative subtype, with promising results observed when delivered as monotherapy or in combination with conventional treatments. In this review, we will report the results of the most recent studies evaluating immune checkpoint inhibitors in breast cancer. In addition, we will discuss the concomitant development of possible biomarkers, which is required for improving the selection of patients with the highest probability of benefiting from these agents.

## 1. Introduction

Breast cancer (BC) is the most common cancer among women worldwide, representing more than 2 million new cases and 600,000 deaths in 2018 [1]. While an increasing number of patients may be cured by a combination of local treatments including surgery, radiotherapy and systemic therapeutics, 5 to more than 11% of patients [2,3,4] present with metastatic disease, and a significant fraction of early BC patients have a micro-metastatic disease resistant to systemic treatment and will ultimately experience a distant relapse, which is still considered an incurable disease [5].

Consistent with its well-known variability in clinical behaviors, BC is a highly heterogeneous disease, composed of different molecular subtypes, which are essentially approximated in the routine setting by combining the expression of the human epidermal growth factor receptor 2 (HER2), estrogen receptor (ER), and progesterone receptor [6,7]. These subtypes, namely HER2 (HER2-positive), luminal (hormone receptor-positive), and triple-negative are associated with diverse clinical outcomes and subtype-driven treatments. HER2-positive BC patients benefit from anti-HER2 treatments, while luminal subtypes require endocrine therapy. Yet, apart from PARP inhibitors for *BRCA-mutated* patients with advanced BC, cytotoxic agents are the gold standard for unselected triple-negative BC (TNBC) patients.

**Figure 1:**
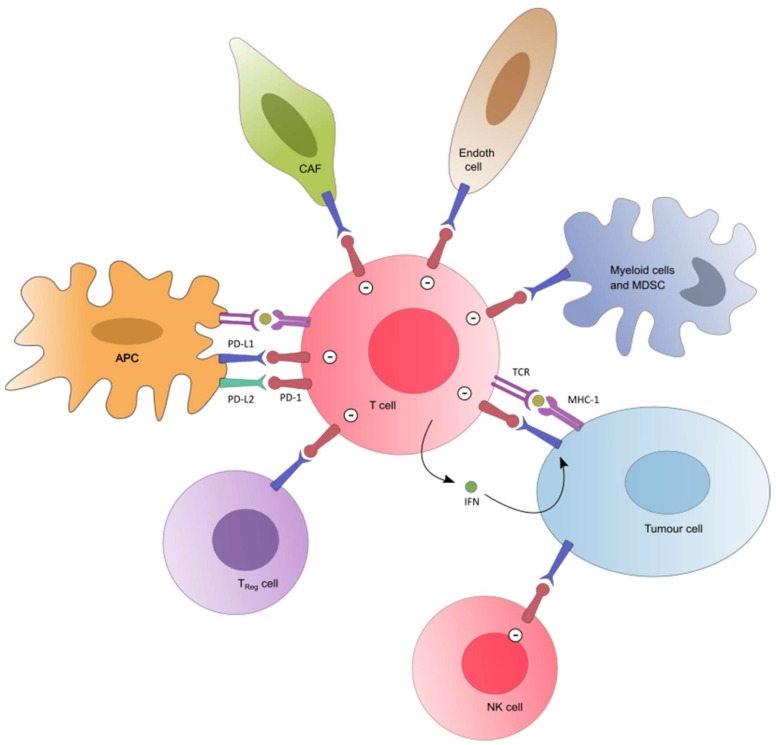
The PD-1/PD-L1 pathway. PD-L2 (green) is expressed in antigen-presenting cells. PD-L1 (blue) is also expressed in tumor cells and in several immune cells (myeloid cells, TReg, endothelial cells). PD-L1 and PD-L2 inhibit T cells and NK cells (minus sign). IFNγ mediates the up-regulation of tumor PD-L1. Abbreviations: APC = antigen-presenting cell; MDSC = Myeloid-derived suppressor cell; NK cell = Natural Killer cell; TReg = Regulatory T cell; CAF = Cancer associated Fibroblast; Endoth cell = Endothelial cell; PD-1 = Programmed cell death-1; PD-L1/2 = Programmed cell death 1 ligand 1/2; TCR = T Cell Receptor; MHC = Major Histocompatibility Complex.

The critical importance of an effective immune system in controlling neoplastic transformation and progression has been described for a long time. Thus, a large body of evidence shows a correlation between a favorable outcome in various malignancies and tumor-infiltrating lymphocytes (TILs) in tumor tissue [5,6,7,8]. Specifically, the presence of CD8+ T-cells and the ratio of CD8+ effector T-cells/FoxP3+ regulatory T-cells seems to correlate positively with an improved prognosis and long-term survival in many solid tumors. The role of programmed cell death 1 (PD-1) receptor-ligand (PD-L1 or PD-L2) interaction was highlighted as a major inhibitor pathway which may be hijacked by tumors to suppress immune control [8,9,10,11,12]. When PD-1 ligands bind to PD-1, T-cell activation through the T-cell receptor is inhibited. PD-L1, which is the PD-1 ligand predominantly involved in negatively regulating the T-cell function in peripheral tissue, may be expressed in various cancers (see recent reviews [13,14] for details regarding methods of measurement and the prevalence in most frequent tumor primaries). Accordingly, disrupting this regulating system has become one of the most attractive therapeutic targets in the immunotherapy of cancers for the last 10 years (Figure 1).

While immunotherapies have improved the prognosis of various cancers (e.g., melanoma, non-small-cell lung cancer, clear cell kidney carcinoma) [15,16,17,18,19], BC has been classically considered as a less immune-sensitive disease [20,21,22]. The reasons for this “resistance” may be related to the few somatic mutation prevalences found in BC (around 1/Mb vs. 10/Mb for melanoma or lung cancer) [23]. Indeed, Yarchoan et al. described a significant correlation between the tumor mutational burden and the objective response rate to PD-1 inhibition (*p* < 0.001) [24]. Furthermore, aggressive BCs are particularly enriched with activated Treg cells with a potent suppressor function [25]. These Treg cells may be enhanced by plasmacytoid dendritic cells with an impaired Interferon (IFN) production [26] Finally, immunotherapy has a best response in ‘inflamed’ tumors (rich in dendritic cells and CD8 T cells) but the proportion of breast cancers that could be considered as ‘inflamed’ tumors is relatively small compared to other diseases and varies substantially between subtypes [27].

However, there is a rationale to support immune-based approaches. First, major survival improvements were achieved in HER2-positive breast cancer with the use of monoclonal antibodies targeting HER2, such as trastuzumab, pertuzumab and trastuzumab emtansine [28,29,30], and their mechanisms of action may at least partially involve the immune system. Second, several immune response-related variables have a significant prognostic value in terms of survival and may be predictive of a response to chemotherapy. For instance, TILs have a positive prognostic impact in survival and predict a high probability of a pathological response to neoadjuvant chemotherapy [31,32,33,34,35], and gene expression signatures of immune response (notably for ER-negative, highly proliferative tumors) were associated with a favorable outcome in TNBC [35,36,37,38,39,40,41,42]. Yet, the impact of TILs on the outcome may be dependent on the subtype, according to a recent study suggesting a poor prognosis associated with TILs in ER+/HER2− BC treated with neoadjuvant chemotherapy [43]. Third, PD-L1 is expressed in BC, which correlates with the presence of TILs, younger age, high grade, lack of ER, overexpression of HER2, TNBC clinical subtypes, as well as basal-like and HER2-enriched molecular subtypes [44]. In addition, PD-L1 expression was associated with a higher pathological response rate to neoadjuvant chemotherapy and a good survival outcome, indicating a likely robust and effective immune response in the tumor microenvironment [44,45].

Altogether, these elements have provided a basis to investigate the role of PD-1/PD-L1-based therapeutics in BC. Regarding the higher expression of TILs and PD-L1 in TNBC, as well as their favorable prognostic and predictive impact in this context, most clinical investigations of PD-1/PD-L1 targeting have been conducted in these specific subtypes, the preliminary promising results of which were recently reported. Yet, some data are also available in other forms of breast cancer, including HER2-positive and luminal cancers. In this article we will provide an overview of the first clinical investigations of PD-1/PD-L1-based therapeutics in breast cancer, from early phase trials to preliminary evidences coming from a recent comparative trial.

## 2. Anti-PD-1/PD-L1 Agents in Breast Cancer: Monotherapy

### 2.1. Pembrolizumab

Pembrolizumab is a humanized monoclonal IgG4-k antibody with a high affinity and selectivity against PD-1, which is currently approved by FDA and/or EMA in a large number of malignancies. 

In KEYNOTE-012 phase Ib [46], pembrolizumab was administered to 32 patients with PD-L1 positive metastatic TNBC. PD-L1 positivity was defined as ≥ 1% on tumor or stroma cells using the 22C3 antihuman PD-1 antibody (Merck & Co., Kenilworth, NJ, USA) and was identified in nearly 60% of the screened patients. There was no limit on prior treatment lines for inclusion. Pembrolizumab was given at a dose of 10 mg/kg until progression or toxicity. The primary endpoint of the study was the objective response rate (ORR). The patient population was heavily pre-treated, with a median number of two pre-treatment lines (range 0 to 9), 100% of patients being already exposed to taxanes, 71% to anthracyclines and 65% to capecitabine. Most patients (78%) had a visceral involvement. Among 27 patients who were evaluable for tumor responses, the ORR was 18.5%, including 1 complete response (CR) and 4 partial responses (PR). The median duration of response was not reached but ranged from 15 weeks to > 47 weeks. The 2-year survival rate was 22%. Of note, the baseline LDH level was associated with a rapid progression of the tumor.

In the KEYNOTE-086 phase II study, two cohorts of metastatic TNBC patients were treated with pembrolizumab 200 mg IV every 3 weeks until a progressive disease, intolerable toxicity, patient withdrawal, or investigator decision and up to 2 years. All patients had a centrally confirmed TNBC, ECOG PS 0-1, LDH < 2.5 ULN, tumor biopsy sample and no radiographic evidence of brain metastases. In Cohort A [47], 170 patients with ≥ 1 prior systemic treatment for metastatic disease and documented progressive disease (PD) were enrolled, 61.8% being PD-L1-positive by immunohistochemistry (IHC), as defined by a combined positive score (CPS)—a ratio between PD-L1-positive cells (tumor or immune cells) and the total number of tumor cells × 100—being ≥1. The ORR was 5.3% (2 complete responses and 7 partial responses) in the overall population, 5.7% in PD-L1-positive patients and 4.7% in PD-L1-negative patients. The disease control rates (DCR, i.e., CR or PR or SD ≥ 24 weeks) were 7.6%, 9.5% and 4.7%, in the total, PD-L1–positive and PD-L1–negative populations, respectively. The median duration of response was 4.4 months in the PD-L1-negative but was not reached in the PD-L1-positive population (6.3–21.5 + months). Median PFS and overall survival (OS) were 2 months and 9 months, respectively, but did not differ between the PD-L1-positive and PD-L1-negative populations. In cohort B [48], 84 patients with no prior systemic treatment and PD-L1 positive tumor were included. The ORR was 24.1%, including 4 CR and 14 PR, with two additional patients reaching an SD for more 24 weeks, leading to a DCR of 23.8%. The median duration of response, PFS and OS were 10.4, 2.1 and 18 months, respectively. Interestingly, stromal TILs in metastatic tissue correlated positively with the ORR in both KEYNOTE-086 cohorts [49]. Of note, poor prognostic factors, such as elevated LDH, a greater number of metastatic sites and the presence of liver involvement, were associated with a lower ORR.

Taken together, these results suggest that pembrolizumab may have actual and durable antitumor activity as a single agent in a limited subset of metastatic TNBC, with a higher probability of success in earlier lines of treatment and in PD-L1-positive patients. The phase III randomized KEYNOTE-119 study compared pembrolizumab to a chemotherapy of the physician’s choice (capecitabine, eribulin, gemcitabine or vinorelbine) in metastatic TNBC pretreated by 1 or 2 lines of chemotherapy, with the stratification (but not selection) based on the expression of PD-L1 by IHC. Enrollment has been completed, and according to a recent press release from Merck the trial did not meet its pre-specified primary endpoint of superior OS compared to chemotherapy. The final results are expected to be presented at an upcoming medical meeting (https://investors.merck.com/news/press-release-details/2019/Merck-Provides-Update-on-Phase-3-KEYNOTE-119-Study-of-KEYTRUDA-pembrolizumab-Monotherapy-in-Previously-Treated-Patients-with-Metastatic-Triple-Negative-Breast-Cancer/default.aspx).

In the large phase I KEYNOTE-028 [50], pembrolizumab was tested in patients with ER+/HER2− tumors. Eligible patients included locally advanced or metastatic disease, after a failure of or inability to receive standard therapy, ECOG PS 0 or 1, ≥ 1 measurable lesion and PD-L1 positivity, using a prototype assay (QualTek Molecular Laboratories, Goleta, CA, USA) and the 22C3 antibody (Merck & Co., Kenilworth, NJ, USA). The specimen was considered to have a positive PD-L1 expression when CPS ≥ 1. Pembrolizumab was administered at 10 mg/kg IV every 2 weeks. A total of 248 patients were screened, and 48 (19.8%) had PD-L1 positive tumors, including 25 heavily pretreated patients (12 had received ≥ 5 lines of prior therapy, including endocrine treatments, for metastatic disease). With a median duration of follow-up of 9.3 months, the ORR was 12% (3 patients), and the clinical benefit rate (as defined as CR + PR + SD for at least 24 weeks) was 20% (5 patients). All responders had been pretreated by at least three lines of chemotherapy. The median duration of response was 12 months (from 7 to 14 months). The safety profile was consistent with previous reports of pembrolizumab in other settings, with 16% of patients experiencing treatment-related grade 3–4 events, and 20% of patients having immune-related events such as grade 1 pneumonitis, grade 2 hypothyroidism, hyperthyroidism and infusion related reactions, and grade 3 hepatitis. Thus, even though it must be judged as being modest, there is a detectable activity of pembrolizumab in pre-treated PD-L1-positive luminal breast cancers.

### 2.2. Atezolizumab

Atezolizumab is an engineered and humanized monoclonal antibody against PD-L1, which stimulates the T cell activity against cancer cells by inhibiting the binding to the PD-L1 receptors PD-1 and B7.1. 

In the first in the human phase 1 study PCD4989g, the activity of atezolizumab in various advanced malignancies was investigated, including TNBC [51]. A total of 116 patients with metastatic TNBC were enrolled (115 with an evaluable disease), with or without prior treatment for metastatic disease, with and without PL-L1 expression by IHC. Atezolizumab was administered every 3 weeks (15 or 20 mg/kg IV or a flat dose of 1200 mg) for a total of 16 cycles or 1 year, with a subsequent amendment making it possible to retreat patients who had discontinued treatment. Treatment beyond the progression was allowed. The primary endpoints were safety and tolerability: treatment-related adverse events (AEs) were frequent (73%) but usually with a grade 1–2 (in 79% of cases), and they included pyrexia, fatigue and nausea, diarrhea, asthenia, and pruritus as the most frequent events, as already seen with atezolizumab in other indications. Grade 3–4 events included pruritic rash, lichen planus, and adrenal insufficiency, pneumonitis, hyperglycemia and pulmonary hypertension. In the overall population, the ORR and DCR by RECIST were 10% and 13%, respectively, but the outcomes were highly dependent on the pre-treatment status (ORR and DCR of 24% and 29%, respectively, in first-line treated patients; 6% vs. 10%, respectively, in patients treated beyond first-line) and on PD-L1 expression by infiltrating immune cells (ORR and DCR of 12% and 15%, respectively, in 91 PD-L1-positive patients, as defined as at least 1% of immune cells stained by an Ventana SP142 IHC assay [VentanaMedicalSystems]; 0% vs. 5%, respectively, in 18 PD-L1-negative patients). The median duration of response was 21 months (range, 3 to ≥38). OS was 8.9 months, but 17.9 months and 10.1 months, in first-line treated or PD-L1-positive patients, respectively, and 7.3 months vs. 6 months in pre-treated or PD-L1-negative patients. Achieving an objective response was associated with a high probability (>80%) of surviving. A high-level of infiltrating immune cells (>10%) was also associated with a better survival. Of note, liver metastases, LDH levels, tumor burden and performance status were associated with worse outcomes.

Thus, and similarly to pembrolizumab, atezolizumab demonstrated proofs of antitumor activity in a subset of TNBC patients, notably when the disease was treated in first-line setting and expressed PD-L1.

### 2.3. Avelumab 

Avelumab is a human anti-PD-L1 IgG1 monoclonal antibody inhibiting the interaction between PD-1 and PD-L1 but not PD-1/PD-L2. Unlike other antibodies disrupting the PD-1/PD-L1 system, avelumab displays some antibody-dependent cell-mediated cytotoxicity (ADCC), which may represent an additional component of its mechanism of action. In the JAVELIN phase Ib study, a cohort of 168 metastatic BC patients, including 58 TNBC, was treated with avelumab 10 mg/kg IV every 2 weeks [52]. All BC subtypes with a measurable disease were eligible. Patients had to be refractory or progressive after the standard of care therapy, but exposed to less than four prior lines of cytotoxic therapy, and, unless contraindicated, had to have received prior treatment with a taxane and anthracycline in any therapeutic setting. A recent tumor tissue sample had to be available, but there was no requirement for PD-L1 expression. Safety was consistent with previous clinical evaluations in other settings, with frequent treatment-related adverse events (68% of patients) but rare AEs ≥ 3 (13%). The most frequent AEs were fatigue, infusion-related reaction and nausea. Immune-related AEs included hypothyroidism, hepatitis and pneumonitis, thrombocytopenia and antinuclear antibody production, dry eye, elevated rheumatoid factor, hyperthyroidism, and pemphigoid skin reaction. Only 4 patients had a grade 3 or more adverse event, including hepatitis and pneumonitis. Antitumor activity was modest, with an ORR in the overall population of 3%, which was slightly higher in TNBC (ORR = 5.2%). A possible explanation for this was a high percentage of pre-treated patients (more than 50% of patients with three prior lines). Of note, in evaluable patients with an available expression of PD-L1 tumor-associated immune cells (10% staining cutoff using PD-L1 IHC 73-10 pharmDx; Dako, Carpinteria, CA, USA), the ORR was 16.7% (and 22.2% [2 of 9 patients] in TNBC) in PD-L1-positive *vs.* 1.6% (2.6% [1 of 39 patients] in TNBC) in PD-L1-negative.

## 3. Anti-PD-1/PD-L1 Agents in Breast Cancer: Combination with Chemotherapy

There are several preclinical evidences supporting a synergism between chemotherapy and anti-PD-1/PD-L1 agents. Thus, an immunogenic cell death has been described following the exposure to various cytotoxic drugs, which may activate dendritic cells and boost the presentation of tumor-associated antigens, ultimately leading to an improved antitumor T-cell cytotoxicity [53,54]. Chemotherapy was also shown to increase the immune infiltrate, particularly in breast cancer [55]. Moreover, some DNA-interacting drugs, such as cisplatin and etoposide, are able to produce a cytosolic leakage of DNA from the nucleus, which was shown to activate the cyclic GMP-AMP synthase (cGAS)/Stimulator of the interferon genes (STING) pathway [56]. The cGAS/STING may stimulate a Type I interferon response and other pro-inflammatory cytokines, leading to an increased antitumor immunity. In addition, various chemotherapeutic agents may inhibit immunosuppressive components in the microenvironment, such as myeloid-derived suppressive cells and Tregs, thereby contributing to reinstate the immune response [57,58,59]. Thus, a combination of cytotoxic chemotherapy and anti-PD-1-PD-L1 agents in breast cancer patients has been evaluated in various settings, including both advanced and early stages.

### 3.1. Advanced Cancers

#### 3.1.1. Early Phase Studies

Pembrolizumab was associated with eribulin in a multicenter, single-arm, open-label, phase Ib/II study (ENHANCE-1/study 218/KEYNOTE-150) aiming to examine the safety and activity of the combination in patients with metastatic TNBC [60]. Eribulin is an anti-microtubule agent with registration in previously treated metastatic BC. Patients could have been previously treated with 0 to 2 lines of chemotherapy for metastatic disease. A total of 107 patients, 106 of them being evaluable, were enrolled, regardless of their PD-L1 status. The ORR was 26.4% (3 patients with a CR and 25 patients with a PR), and the CBR was 32.8%. Of note, the ORRs were not significantly different regarding the PD-L1 status (30% in PD-L1-positive vs. 22% in PD-L1-negative; out of the 3 patients who experienced a complete response, 1 patient was PD-L1 negative) or prior chemotherapy exposure (29% in untreated patients vs. 22% in patients with 1–2 previous lines). These results compare favorably with those obtained with the eribulin single agent. The duration of responses was long (median of 8.3 months, lasting more than 6 months in 53% of responders), and the median PFS and OS were 4.2 and 17.7 months, respectively. Treatment-related AEs of the combination were comparable to those observed with each treatment as a monotherapy. The most common adverse events were asthenia (73.6%), nausea (51.2%), peripheral sensory neuropathy (46.3%), alopecia (43.9%) and pyrexia (36.6%). AEs of grade 3–4 related to pembrolizumab were observed in 19.5% of patients. Thus, a combination of eribulin and pembrolizumab was well tolerated and demonstrated an antitumor activity in patients with metastatic TNBC.

In the GP28328 multicenter, multicohort, phase 1b study, atezolizumab was combined with chemotherapy in the treatment of various advanced solid tumors. In a cohort of metastatic TNBC patients, atezolizumab was tested in association with nab-paclitaxel [61]. Nab-paclitaxel, a nanoparticle albumin-bound form of paclitaxel with registration in the treatment of advanced breast cancer, was selected because of the lack of requirement for steroid premedication, which could impede the antitumor activity of immune-oncology agents and because of the potential of taxanes to stimulate an anticancer immune response. Patients were eligible if they had a good performance status, archived or freshly collected tumor specimens, no more than two prior systemic cytotoxic regimens for advanced disease, no taxane exposure within the last six months, and no active central nervous system disease. A total of 33 patients were enrolled and received IV atezolizumab 800 mg on days 1 and 15, plus nab paclitaxel, 125 mg/m^2^, on days 1, 8, and 15 of each 28-day cycle, including a 25-patient subgroup with a research serial biopsy in which atezolizumab was started at day 15 of cycle 1. The primary endpoint was safety and tolerability, while the secondary endpoints included the ORR by RECIST 1.1, the duration of response, DCR, PFS, OS and biomarkers analyses. Twenty patients were treated in a second-line setting or later, and nearly 90% of patients had been previously exposed to taxanes. The ORR was 39.4%, and the median duration of response was 9.1 months. The DCR was 51.5%, and the median PFS and OS were 5.5 months and 14.7 months, respectively. The ORR did not differ significantly according to the pre-treatment status, but was numerically higher for the first-line treated versus the second-line or later (53.8% vs. 30%) and in PD-L1-positive vs. PD-L1-negative (41.4% vs. 33.3%), the PD-L1 expression being defined as the staining of at least 1% of immune cells using the VENTANA PD-L1 (SP142) assay (Ventana Medical Systems, Inc, Oro Valley, AZ, USA.,). The serial tumor biopsy study indicated that there was no significant change in the PD-L1-positive immune cells, CD8+ T cells, or stromal TILs following the treatment exposure. Yet, as already observed in the single agent studies, the atezolizumab plus nab-paclitaxel treatment was associated with a detectable increase in activated proliferating CD8+ T cells in the peripheral blood and *CXCL10* RNA expression in peripheral blood mononuclear cells, suggesting that concomitant chemotherapy does not affect the effect of atezolizumab on immune stimulation. The tolerance profile included expected AEs from chemotherapy such as neutropenia, fatigue, alopecia, diarrhea, and peripheral neuropathy, as well as immune-related side effects such as rash, pruritus, increased transaminases, dry skin and pyrexia, reaching a grade 3–4 in 21% of patients. Atezolizumab was discontinued due to toxicity in 3 patients (2 for pneumonitis). Thus, combining nab-paclitaxel and atezolizumab was feasible with a promising anti-tumor activity and a manageable toxicity profile.

In a phase Ib study enrolling 28 metastatic TNBC patients, pembrolizumab (200 mg IV every 21 days) was evaluated as a first-line treatment in association with either paclitaxel (80 mg/m² weekly) or capecitabine (2000 mg BID 7 days on/7 days off), according to the physician’s choice [62]. Both combinations were considered as being tolerable. In capecitabine-treated and paclitaxel-treated patients, the ORR was 43% (5 PR, 1 CR, 2 SD) and 25% (1 CR, 1 PR, 3 SD), respectively. Subjects with a rapid progression (less than 12 months) from initial therapy had a lower response rate. Of note, a decline in the T cell quantity was detected during treatment with both agents.

Pembrolizumab was also evaluated in combination with capecitabine in a mixed population of HER2-negative (either TNBC or endocrine-refractory hormone receptor-positive) metastatic breast cancer patients in a single-arm phase II study [63]. Patients received pembrolizumab 200 mg IV D1 and capecitabine 1000 mg/m² BID D1-14 of a 21-day cycle. In 29 evaluable patients, the median PFS was 4.1 months and median OS was 15.4 months, whereas the ORR was 14% (*n* = 4), SD was 41% (*n* = 12), and CBR was 28% (*n* = 8). The results were similar in TNBC and luminal subtypes and did not differ from what was expected from the historical series of capecitabine alone. Toxicities were mostly moderate and consistent with classical side effects of both agents, but there was one death (hepatic failure in a patient with liver metastases), which could be possibly related to pembrolizumab.

#### 3.1.2. Randomized Studies

An adaptive phase II study (TONIC trial) using a non-comparative Simon’s two stage design was conducted to evaluate different schedules of short-term (2-week), low-dose, cytotoxic induction (2 × doxorubicin 15 mg weekly vs. cyclophosphamide 50 mg daily orally vs. 2 × cisplatin 40 mg/m^2^ weekly) or no induction treatment before the administration of nivolumab monotherapy [64]. While low-dose metronomic cyclophosphamide may deplete immune-suppressive Tregs, cisplatin could up-regulate MHC-1 molecules and stimulate the T-cells activity, and doxorubicin may induce an immunogenic cell death, all being potentially synergistic with anti-PD-1. An additional arm assessed the prior irradiation of one metastatic lesion (3 × 8 Gy), which may also activate the STING/Interferon Type 1 pathway and prime T-cells. Eligible patients were metastatic TNBC pretreated with a maximum of three lines of palliative chemotherapy. The preliminary results of the stage I phase (*n* = 67) identified doxorubicin (ORR = 35%, *n* = 17) and cisplatin (ORR = 23%, *n* = 13) as the more efficient induction strategies. The evaluation of post-nivolumab biopsies identified more T-cells infiltration and more clonal T-cells (as identified by TCR sequencing) in responding patients.

In a randomized phase II study, eribulin with or without pembrolizumab was evaluated in 88 hormone receptor-positive/HER2-negative metastatic breast cancer patients [65]. Patients had to be pretreated by at least 2 lines of endocrine therapy or appropriate candidates for front-line chemotherapy and could have received 0 to 2 lines of chemotherapy for advanced disease. No selection was made on PD-L1 expression, but archival tumor tissue was to be available before inclusion. Patients were randomized 1/1 to receive eribulin 1.4 mg/m² D1-D8 every 21 days plus or minus pembrolizumab 200 mg IV D1. A cross over to single-agent pembrolizumab was allowed for control arm patients in the case of a progressive disease. More than 50% of patients had received previous chemotherapy for an advanced disease (54% in pembrolizumab versus 67% in the control arm). The median PFS were similar (4.2 in pembrolizumab versus 4.1 months in control, stratified HR = 0.8, 95% CI: 0.5–1.3, *p* = 0.33), and there was no difference in the ORR, CBR, duration of response and OS. Archival tumor tissues (primary or metastatic in 69% and 31% of cases, respectively) were tested for the expression of PD-L1 (IHC using a 22C3 antibody and considered positive if ≥ 1% of staining in the tumor cells or mononuclear inflammatory cells). PD-L1 positivity was found in 29% (pembrolizumab) and 25% (control) of patients, but there was no trend for the efficacy of pembrolizumab in this small subgroup of patients. Patients receiving single-agent pembrolizumab after a cross-over did not experience any objective response. Of note, 2 patients experienced treatment-related death (colitis, in the context of neutropenia and sepsis in both patients).

In the Impassion-130 randomized phase III study, first-line treated metastatic TNBC patients with a good performance status (0–1) were randomized to receive weekly nab-paclitaxel (100 mg/m² D1, D8, D15) plus atezolizumab (800 mg D1, D15) or a placebo, every 28-day cycle [66]. Radiation therapy and previous chemotherapy, including taxanes, were permitted but had to be delivered at least 12 months before the randomization. Patients with asymptomatic and treated CNS metastases were eligible. Patients were stratified according to the presence of liver metastases, prior taxanes in the adjuvant and/or neoadjuvant setting, and the expression of PD-L1 on the tumor infiltrating immune cells (<1% vs. at least 1%) by IHC using the above-described Ventana assay. The primary endpoint was PFS, with a subsequent amendment to include OS as a co-primary. Both endpoints had to be sequentially tested in the intention-to treat population and in the PD-L1–positive subgroup. A total of 902 patients were included (451 patients in each group), including 369 patients (40%) with a PD-L1-positive tumor. Approximately 25% of patients had liver metastases, and 63% of patients had received a prior adjuvant/neoadjuvant treatment, including 51% of patients with a prior taxanes exposure. With a median follow-up of 12.9 months, atezolizumab marginally but significantly increased PFS in the overall population (median of 7.2 months vs. 5.5 months, HR = 0.80, 95% CI [0.69–0.92], *p* = 0.002). However, in the PD-L1-positive subgroup of patients, the increase in PFS was more substantial and clinically relevant (median of 7.5 months vs. 5 months, HR = 0.62, 95% CI [0.49–0.78], *p* < 0.001). At the time of the first OS interim analysis (median follow-up = 12. months), with less than 50% of survival events, OS was not significantly different between atezolizumab and the placebo in the overall population (median of 23 months vs. 17.6 months, HR = 0.84, 95% CI [0.69–1.02], *p* = 0.08). However, in the PD-L1-positive subset of patients, a large and clinically meaningful numerical improvement in OS was observed in the atezolizumab arm (median of 25 months vs. 15.5 months, HR = 0.62, 95% CI [0.45–0.86]). However, as per protocol, the statistical significance could not be tested in this subgroup, since the OS improvement was not confirmed in the whole population at this time. Of note, in a recently reported update (second interim analysis after a median follow-up of 18 months), the median OS was still not significantly different between each arm (21 months versus 18.7 months, stratified HR = 0.86, *p* = 0.07) in the whole population, and the numerical difference in OS in the PD-L1-positive subset tended to decrease (median OS of 25 months versus 18 months, HR = 0.71, no formal *p*-value by protocol design) [67].

Other efficacy variables were also in favor of atezolizumab: the ORR was significantly increased (from 45.9 to 56%, *p* = 0.002 and from 42.8 to 56.9%, in the overall and PD-L1-positive patient populations, respectively), including a numerical increase in the complete responses (from 1.6 to 7.1% and from 1.1% to 10.3%, in the overall and PD-L1-positive patient populations, respectively) and the durations of the responses were increased (from 5.6 to 7.4 months and from 5.5 to 8.5 months, in the overall population and in the PD-L1-positive subgroup, respectively). Regarding safety, nausea, cough, neutropenia, pyrexia, and hypothyroidism were most frequent in the atezolizumab arm, as was the rate of grade 3–4 events (the most frequent of which being neutropenia, a decreased neutrophil count, peripheral neuropathy, fatigue, and anemia). Potentially immune-related events reaching a grade 3–4 were observed in 7.5% of the atezolizumab-treated patients vs. 4.3% in the placebo arm. Immune-related hypothyroidism was noted in 17.3% of atezolizumab-treated patients (vs. 4.3% in the placebo arm). A limited number of pneumonitis was observed (in 3.1% of patients receiving atezolizumab vs. 0.2% in those that were placebo-treated). Toxic deaths were noted in three atezolizumab-treated patients (autoimmune hepatitis, mucosal inflammation, and septic shock) vs. 1 (hepatic failure) in placebo-treated patients. Treatment (either atezolizumab or nab-paclitaxel) was discontinued due to AEs in 15.9% of patients receiving atezolizumab vs. 8.2% in the placebo arm. Based on these results, FDA has recently approved atezolizumab in adult patients with unresectable locally advanced or metastatic TNBC whose tumors express PD-L1, as determined by an IHC based on the VENTANA assay which was also FDA-approved. Of note, this indication was approved under accelerated approval, but the continued approval for this indication may be conditional and subject to a re-challenge according to the verification and description of the clinical benefit in confirmatory trials. Accordingly, atezolizumab is also being tested as a first-line treatment in combination with other chemotherapy regimens in ongoing or recently completed trials, including:

- Weekly paclitaxel in the Impassion 131 study, which enrolled a similar patient population as in Impassion 130 and is under analysis.
- Carboplatin-gemcitabine or capecitabine in the Impassion 132 study, which is dedicated to patients with an early relapse (<12 months) after anthracyclines/taxanes administered in the adjuvant/neoadjuvant setting and which is ongoing.

Of note, in the recently completed KEYNOTE-355 study, first-line treated patients with metastatic or locally recurrent inoperable TNBC were randomized between chemotherapy plus pembrolizumab vs. chemotherapy plus placebo. Chemotherapy could be weekly paclitaxel, weekly nab-paclitaxel or carboplatin-gemcitabine, according to the physician’s choice. Patients with prior adjuvant/neoadjuvant chemotherapy were eligible if it was completed at least 6 months before the randomization. The patient population was stratified according to the PD-L1 status by IHC, and the primary endpoints included both PFS and OS in all patients, as well as in the PD-L1-positive subgroup.

In the ongoing SAFIR 02 study, HER2-negative, either TNBC or ER+/endocrine-resistant metastatic breast cancer patients receive first- or second-line cytotoxic treatment, and patients with a stable disease or objective response after a minimum of 4 and a maximum of 8 cycles of chemotherapy and without actionable molecular alterations are randomized between chemotherapy continuation or immunotherapy with durvalumab (a human, IgG1 anti-PD-L1 monoclonal antibody) as maintenance [68].

### 3.2. Early Settings

In the I-SPY 2 trial, an adaptive randomized phase II trial evaluating the combination of various targeted or biological drugs with a conventional chemotherapy backbone (sequential association of paclitaxel for 12 weeks, followed with 4 cycles AC) in the neoadjuvant setting, the association of 4 cycles of pembrolizumab at 200 mg 3 times weekly to paclitaxel was tested in HER2-negative subtypes [69]. Sixty-nine patients were enrolled, and in 29 TNBC patients, pembrolizumab increased the estimated pCR rates from 20% to 60%. Of note, in 40 patients with high-risk ER+/HER2− patients, the estimated pCR was also improved from 13% to 34%.

In the KEYNOTE-173 phase Ib study, 60 patients with locally advanced TNBC have been enrolled and received a neoadjuvant treatment with pembrolizumab, associated with different schedules of chemotherapy [70]. One cycle of pembrolizumab alone was first administered 21 days before initiating the combination for 8 additional cycles. The cytotoxic regimen included weekly nab-paclitaxel at 125 mg/m^2^ (cohort A), weekly nab-paclitaxel at 100 mg/m^2^ plus 3-weekly carboplatin AUC6 (cohort B), weekly nab-paclitaxel at 125 mg/m^2^ plus 3-weekly carboplatin AUC5 (cohort C), weekly nab-paclitaxel at 125 mg/m^2^ plus weekly carboplatin AUC2 (cohort D), weekly paclitaxel at 80 mg/m^2^ plus 3-weekly carboplatin AUC5 (cohort E), and weekly paclitaxel at 80 mg/m^2^ plus weekly carboplatin AUC2 (cohort F), followed by 3-weekly doxorubicin at 60 mg/m^2^ and cyclophosphamide at 600 mg/m^2^ prior to surgery. 60 patients have been enrolled, and the overall pCR rate (ypT0/Tis ypN0) was 60%. Grade 3 or more adverse events were common and included neutropenia (73%), febrile neutropenia (22%), anemia (20%) and thrombocytopenia (8%). Most common immune-related AEs were grade 2 hypothyroidism (4 patients), grade 1 hyperthyroidism (3 patients), grade 3 colitis (2 patients), and grade 3 rash (2 patients), leading to a pembrolizumab discontinuation in 11 patients.

In the GeparNuevo randomized phase II trial, 174 patients with TNBC were randomized to receive either durvalumab at 1.5 g or a placebo every 4 weeks in combination with weekly nab-paclitaxel for 12 weeks, followed with dose-dense EC for 4 cycles [71]. Initially a “window of opportunity” sequence was scheduled, in which patients received durvalumab at 0.75 g or a placebo for 2 weeks before biopsy and chemotherapy, plus durvalumab or a placebo combination. Patients were stratified according to the stromal TILs abundance. The pCR was numerically higher in durvalumab-treated patients (53.4% vs. 44.2% in placebo-treated patients), but the difference was not statistically significant. Interestingly, the difference was more pronounced in favor of durvalumab in the subset of patients receiving an initial 2-week single-agent “window” treatment (61% vs. 41.4%), but not present in patients without the “window”. The benefit was also more prominent in patients with stage IIa and higher (55.4% vs. 38.6%) and in younger patients (<40 years, 69.2% vs. 42.9%). A large biomarker analysis was performed and showed that the presence of stromal TILs was associated with pCR in both treatment groups, while intra-tumoral TILs at the baseline biopsy did not predict either pCR or any treatment effect. Interestingly, an increase in intra-tumoral TILs during the window phase was observed in both treatment arms, which predicted pCR only in durvalumab-treated patients. Regarding PD-L1, nearly 90% of the available samples (138 out of 158) were PD-L1-positive (either PD-L1-positive on the tumor cells or on the immune cells). The pCR rate was higher in patients with PD-L1-positive tumors in both treatment arms, the effect being statistically significant for tumor cell PD-L1 expression in the durvalumab arm and for immune cell PD-L1 expression in the placebo arm [72].

The tolerance was similar between arms, except for hypo/hyperthyroidism, which was higher in the durvalumab arm. A phase III randomized study is ongoing and will include a run-in phase with single-agent durvalumab.

## 4. Anti-PD-1/PD-L1 in Breast Cancer: Combination with Targeted Therapies

### 4.1. Combination of Anti PD-1/PD-L1 Agents with PARP Inhibitors

Breast cancer patients carrying germline *BRCA1* or *BRCA2* mutations represents around 5% of cases. While most cancers with *BRCA1* mutation are TNBC, cancers associated with *BRCA2* may display all subtypes of breast cancer, with a similar frequency as in sporadic subtypes. Breast cancers associated with *BRCA1/2* mutations have a deficiency in homologous recombination repair, a mechanism of DNA double-strand breaks repair, the defect of which is synergistically lethal with the inhibition of single-strand DNA repair [73]. Poly(ADP-ribose) polymerase (PARP) plays a major role in single-strand DNA repair, and PARP inhibitors have demonstrated antitumor activity in HER2-negative metastatic breast cancer patients, associated with *BRCA1/2* germline mutations. In the randomized phase III trials, the PARP inhibitors olaparib and talazoparib both significantly improve PFS over a chemotherapy of the physician’s choice, in HER2-negative metastatic breast cancer patients with a prior exposure to anthracyclines, taxanes and endocrine therapy if ER+ [74,75].

Previous translational investigations have suggested an increase in stromal TILs and a higher tumor mutational burden in *BRCA1* mutation-associated breast cancer [76]. In a preclinical model of *BRCA1*-mutated breast cancer, the combination of a DNA-damaging agent, such as cisplatin, and immune checkpoint inhibitors (anti-CTLA4 and/or anti-PD-1) was found to be synergistic. In addition, preclinical works on breast cancer cell lines and xenografts found that PARP inhibitors may up-regulate PD-L1 expression in cancer cells, which inhibits T-cell cytotoxicity. Combining PARP inhibitors and anti-PD-L1 was found to be synergistic with PARP inhibitors, both in proficient and defective *BRCA* models [77].

In the phase II open label multi-arm MEDIOLA study, 34 metastatic patients with germline *BRCA*1/2 mutations received a combination of olaparib and durvalumab, after a run-in sequence with single-agent olaparib for 4 weeks. The tumor biopsy was obtained at baseline and after initial single-agent olaparib [78]. Olaparib was administered orally at 300 mg twice daily continuously, and durvalumab was given intravenously at 1.5 g every 4 weeks. In the 30 patients evaluable for efficacy, the disease control rate (primary endpoint of the study) was 80% (24 of 30 patients) at 3 months in the entire study population. The ORR at 28 weeks was 63% (95% CI, 44–80%), the median duration of response was 9.2 months (range: 5.5–13.1), and the median PFS was 8.2 months (95% CI, 4.6–11.8) at 28 weeks. The disease control rate was 50% (90% CI, 34–66%) at 28 weeks. The efficacy seemed to be dependent on the extent of the prior treatment: the median duration of response in patients with 0 or 1 line of prior chemotherapy (*n* = 14) was 12.9 months, vs. 5.5 months in those with 2 prior lines of chemotherapy (*n* = 5). The median PFS was 11.7 months (95% CI, 4.57–13.77) in patients treated with 0 or 1 prior line of chemotherapy and 6.5 months (95% CI, 0.99–8.25) in those treated with 2 lines. There was a correlation between PD-L1 expression at baseline or after olaparib monotherapy, T-cells, CD8+ T-cells and the response.

In the TOPACIO/KEYNOTE-162 trial, unselected metastatic TNBC patients with a progressive disease after adjuvant/neoadjuvant chemotherapy, and with no more than 2 lines of chemotherapy for advanced disease (including platinum, provided that no progression was observed while on or within 8 weeks of the last platinum), received oral niraparib at 200 mg/day continuously in combination with IV pembrolizumab at 200 mg every 3 weeks [79]. Fifty-four patients were enrolled, including 15 patients with a *BRCA* mutation. Overall, the ORR was 28% and DCR was 50%. In *BRCA* mutation carriers, the ORR and DCR were higher (60% and 80%, respectively). The main toxicities were hematological, as expected, with PARP inhibitors, but no new safety signal was identified.

#### 4.1.1. Combination of PD-1/PD-L1 Inhibitors with Trastuzumab

A large number of TILs may be identified in primary HER2-positive BC, the presence of which predicts pCR and a favorable survival outcome [80]. The mechanisms of action of trastuzumab include some immune-based processes [81,82], including the recruitment of immune effectors by Fc fragment and antibody-dependent cellular cytotoxicity, and preclinical studies have shown that the association of trastuzumab with anti-PD-1/PD-L1 immune checkpoint inhibitors may be synergistic and may be effective in the case of trastuzumab resistance [83]. In the PANACEA phase 1b/2 trial, HER2-positive breast cancer with a documented progression during previous trastuzumab-based therapy, a RECIST measurable disease and good PS, received a combination of pembrolizumab and trastuzumab [84]. In the phase 1b, the dose escalation part, two doses of pembrolizumab were used: 2 mg/kg and 10 mg/kg were given every 3 weeks. Trastuzumab was administered at the standard dose (6 mg/kg every 3 weeks). In part 2 of the trial, a flat 200 mg dose of pembrolizumab was used. In phase Ib, all patients had PD-L1-positive tumors, whereas in the phase 2 stage, 40 patients had PD-L1-positive tumors and 12 patients had PD-L1 negative tumors. In the phase 1 part, no dose-limiting toxicities, cardiovascular toxic effects, or deaths was observed, and one CR out of 6 patients was reported, the patient being treated at 2 mg/kg during 35 cycles, before interrupting pembrolizumab due to immune-related gastritis. In addition, another patient treated at 10 mg/kg reached CR but had a CNS progression after cycle 4. The patient was treated by surgery and whole brain radiotherapy and was kept on treatment during 18 months. In the phase 2 PD-L1-positive population (n = 40), 6 patients had a confirmed objective response (15%), and disease control was achieved in ten (25%). The median follow-up was 13.6 months, and the 12-month OS was estimated to be 65%. By contrast, no objective response or disease control was observed in the PD-L1-negative population, and the estimated 12-month overall survival was 12%. By combining the phase 1 and phase 2 PD-L1-positive patients, the median duration of response was 3.5 months and median duration of disease control was 11.1 months. In terms of tolerance, the most common treatment-related AEs were fatigue (21%), diarrhea (14%), and arthralgia (14%). Nearly 30% of patients experienced grade 3 or more treatment-related AEs, including severe events such as pneumonitis, drug-induced liver injury, dyspnea, autoimmune disorder and diarrhea. Nineteen patients had any-grade immune-mediated AEs, including thyroid dysfunction, pneumonitis and autoimmune hepatitis. Treatment discontinuation due to toxicity was necessary in 8% of the patients. Of note, the rate of TILs was examined on pre-treatment metastatic tissue samples and was found to be low (median 1.5%) compared to what is described in primary HER2+ breast cancer. The TIL level was higher in PD-L1 positive tumors, and it increased in objective responders and in patients with disease control. Thus, there are preliminary clinical evidences suggesting an actual antitumor activity of anti PD-1 in selected patients with pre-treated, trastuzumab-resistant advanced HER2+ breast cancer.

#### 4.1.2. Combination of PD-1/PD-L1 Inhibitors with CDK4/6 Inhibitors

Cyclin-dependent kinase 4/6 (CDK4/6) inhibitors have become a major component of systemic treatment in ER+/HER2− advanced breast cancer, in which they are associated with endocrine therapy and may improve outcomes either in hormone-sensitive or insensitive disease [85,86,87,88,89]. Several preclinical data have shown links between cell cycle inhibition with CDK4/6 inhibitors that may induce apoptosis, senescence and inflammation, with the recruitment of immune effectors, including T-cell infiltration, which may participate in antitumor activity [90]. Recently, abemaciclib and other CDK4/6 inhibitors have been shown to enhance antitumor immunity in preclinical models by overcoming two central mechanisms of tumor immune evasion [91,92,93]: first, CDK4/6 inhibitors activate the tumor cell expression of endogenous retroviral elements, thus increasing intracellular levels of double-stranded RNA. This in turn stimulates the production of type III interferons and therefore enhances the tumor antigen presentation; second, CDK4/6 inhibitors markedly suppress the proliferation of regulatory T-cells. Ultimately, these events promote the cytotoxic T-cell-mediated clearance of tumor cells, which may be further enhanced by the addition of an immune checkpoint blockade. Recently, data have been reported from early phase trials evaluating feasibility and preliminary antitumor activity. Thus, a phase Ib trial evaluated the combination of abemaciclib and pembrolizumab in 28 hormone-resistant advanced breast cancer patients, pre-treated with up to two lines of chemotherapy [94]. The maximum tolerated dose was abemaciclib at 150 mg twice daily plus IV administered pembrolizumab at 200 mg on day 1 of each 21-day cycle. A randomized comparative study is ongoing.

#### 4.1.3. Combination of PD-1/PD-L1 Inhibitors with MEK Inhibitors

The preclinical data indicate that the mitogen-activated protein kinase (MAPK) pathway may be involved in immune resistance. Accordingly, targeting the mitogen-activated protein kinase/extracellular signal–regulated kinase kinase (MEK) may be an effective strategy to convert immune-resistant cancers by recruiting immune cells. In spite of initial concerns about the potential for these drugs to alter the T cell function, it has been shown that the treatment of melanoma cell lines with MEK inhibitors was associated with an enhanced melanoma antigen expression and apoptosis in tumor cell lines with an increased expression of HLA molecules, and synergy was demonstrated with the immune checkpoint blockade in preclinical models [95]. This was further illustrated in advanced melanoma patients in which MEK inhibitors were able to increase intra-tumor cytotoxic lymphocytes [96]. In advanced TNBC, the COLET study initially showed an improvement in the ORR for patients receiving an association of cobimetinib (a MEK inhibitor) and paclitaxel [97]. A subsequent cohort of patients in the same study was randomized to receive atezolizumab at 840 mg IV D1-D15 + oral cobimetinib at 60 mg qd D3-23 with either paclitaxel at 80 mg/m² D1-D8-D15 or nab-paclitaxel at 100 mg/m² D1-D8-D15, every 28 days, as a first-line treatment. In 63 patients evaluable for efficacy at the time of the report, 34% (11 out of 32) of those receiving paclitaxel and 29% (9 out of 31) of those receiving nab-paclitaxel achieved an objective response. These results looked similar to those previously reported for cobimetinib plus paclitaxel without atezolizumab, but there was a numerical trend for a higher response rate in subgroups with PD-L1-positive tumor-infiltrating immune cells. The safety profile was consistent with the previously known toxicities of each agent, without evidences for an increase resulting from atezolizumab [98]. Table 1 recapitulates the reported studies evaluating ICI in breast cancer. A large number of breast cancer-focused clinical trials evaluating various combinations between anti-PD-1/PD-L1 inhibitors and other agents are ongoing, as indicated in Table S1.

## 5. Surrogate Markers of PD-1/PD-L1 Inhibitors Efficacy

Due to the cost of immune checkpoint inhibitors (ICI) and the development of this therapeutic class through several cancer types, as well as the potential incidence of immune-related adverse events, the identification of surrogate markers of ICI efficacy is clinically meaningful and required by regulatory authorities. In breast cancer tumor cells (TC), PD-L1 expression is low (around 10%) [99]. Accordingly, and contrary to other tumor types, PD-L1 expression, when measured on tumor cells, is not a valid predictive biomarker of ICI efficacy in BC. As an example, in the Keynote 086 trial, investigating pembrolizumab in patients with previously treated mTNBC, the ORR (95% CI) was 5.3% (2.7–9.9) in the total population and 5.7% (2.4–12.2) for PD-L1^+^ cases [47]. The PD-L1 TC expression was neither a predictor of the response to avelumab (JAVELIN trial) nor to atezolizumab (IMpassion 130) [52,66].

In immune cells (IC), PD-L1 is expressed in CD11b^+^ myeloid cells (dendritic cells and macrophages mainly, but also T cells and NK cells). In the Impassion 130 investigating atezolizumab in combination with nab-paclitaxel for mTNBC, PD-L1 IC expression was a stratification parameter (threshold at 1%, using SP142 antibody, Ventana). The subgroup of patients with PD-L1 > 1% (185/451 patients) benefited particularly from atezolizumab (HR for OS = 0.62; 95% CI 0.45–0.86) compared to the general population (HR for OS = 0.84; 95% CI 0.69–1.02) [66]. Additionally, in the mBC subset of the JAVELIN trial (phase 1b, avelumab), a trend toward a higher ORR was seen in patients with PD-L1 IC^+^ vs. patients with PD-L1 IC^−^ in the overall population (16.7% vs. 1.6%) [100,101]. However, the reproducibility of PD-L1 detection in immune cells is highly questioned by several pathologists. Additionally, in the Blueprint project, Ventana SP142 underperformed for the PDL1 staining of lung cancer samples, compared to three other anti-PD-L1 antibodies [102]. Nevertheless, the FDA recently granted an accelerated approval to atezolizumab in combination with nab-paclitaxel patients with unresectable locally advanced or metastatic triple-negative breast cancer whose tumors express PD-L1 (PD-L1 IC ≥ 1% of the tumor area). This approval is associated with the approval of the VENTANA PD-L1 (SP142) assay as a companion diagnostic assay.

A recent retrospective study examined PD-1 mRNA expression in 10,078 tumor samples representing 34 different cancer types from TCGA and found a significant correlation between PD-1 mRNA and the ORR following anti-PD-1 monotherapy, while PD-L1 tumor expression by IHC or the percentage of TILs were not found to be associated with the response [103].

Among other tumor types, the breast cancer mutation load is in the low average (around 1 mutation per Mb), even if it is more important in ER^-^ breast cancers [104]. Interestingly, TMB-High is associated with improved survival, probably due to a protective immune infiltration and a “hot tumor” phenotype [105]. However, contrary to other tumors types like melanoma [106] and lung cancer, in which recent studies support TMB as a predictive biomarker for ICI efficacy [107,108], TMB was not demonstrated as a predictor of ICI efficacy in breast cancer [109], notably in the IMPassion 130 study [66], but few data are available about TMB and response to immunotherapy in BC. Recently, a cohort of the basket trial TAPUR (Targeted Agent and Profiling Utilization Registry) evaluated heavily pre-treated metastatic breast cancer patients with high TMB receiving pembrolizumab. High TMB was defined as at least 9 mutations per Mb, according to a FoundationOne test or another TAPUR-approved test. The ORR was 21% (6 out of 28), and the disease control rate (PR or SD for at least 16 weeks) was 37%, suggesting a certain level of activity of pembrolizumab in this subset of patients. Recent data focusing on lung cancer, colorectal cancer, or melanoma have suggested that the circulating mutation burden, identified by the massive parallel sequencing of cell-free DNA, may be correlated to the tumor mutation load and to the PD-1 and PD-L1 inhibitors’ efficacy [110,111,112]. Moreover, early changes of circulating tumor DNA levels may be associated with a response to ICI [113]. Similar results have not been described for breast cancer.

Of note, no tumor associated antigens (TAAs) have been shown to be associated with the ICI response. Conversely, a high stromal TIL number was significantly correlated with a better response to the PD-1 inhibitor (pembrolizumab, Keynote 086) when administered as monotherapy in the first-line setting for metastatic TNBC (ORR: 39.1% above the median stromal TIL; ORR: 8.7% below the median stromal TIL) [49]. These data are consistent with Keynote 173, where the pre-treatment and on-treatment TIL levels are associated with higher pCR rates (Loi et al., ESMO Breast Cancer 2019). More specifically, a translational analysis using single cell RNA-seq revealed that a specific subset of T cells (CD8+, resident memory) was significantly associated with improved patient survival in early-stage TNBC [114]. To date, the study of immune infiltrate is mainly focused on T cells, but it is highly probable that other cells types are important in the tumor microenvironment, particularly myeloid cells (macrophages, MDSC).

Finally, beside the two most advanced surrogate biomarkers (PD-L1 IC and stromal TILs), some clinical predictors of treatment efficacy may be used. For example, the number of previous lines of treatment and the number of metastatic sites seem to be associated with resistance to ICI. In the IMPassion 130 trial, the efficacy of the atezolizumab plus nab-paclitaxel combination was higher for previously chemotherapy-naïve (HR for progression or death = 0.72 (95% CI 0.57–0.92)) vs. 0.85 (95% CI 0.71–1.03)) patients who received (neo)adjuvant chemotherapy. Concerning the number of metastatic burdens, HR = 0.76 (95% CI 0.64–0.91) for patients with 0 to 3 metastatic sites vs. 0.89 (95% CI 0.67–1.17) for patients with more than 3 metastatic sites.

## 6. Immune Combination Using PD(L)1 Inhibitors and Future Development in BC Immunotherapy

The combination of anti-CTLA-4 and anti-PD(L)-1 is appealing for two reasons: (i) a biological rationale suggesting a synergy between anti-CTLA-4 (activating the T cells in the lymph node) and anti-PD(L)-1 (activating the T cells in the tumor site); and (ii) the successful ipilimumab/nivolumab association in melanoma [115] and in NSCLCC TMB high patients [107]. In breast cancer, a phase II trial of the ipi/nivo combination (NCT03342417) was terminated in May 2019 for slow patient accrual. However, the ipi/nivo combination is also investigated by the NIMBUS trial (NCT03789110), a phase II sponsored by the Dana Farber, including hypermutated BC (tumor mutational burden of at least 10 mutations per megabase assessed by a cancer-gene panel containing more than 300 genes). The tremelimumab/durvalumab combination is also investigated in the neoadjuvant setting in HR+ HER2− Stage II-III breast cancer (NCT03132467) or in the metastatic setting (NCT02536794 and MOVIE trial NCT03518606).

New ICI combinations are also in development. Antibodies targeting other co-inhibitory molecules, like anti-LAG3 (IMP321 and BMS-986016), anti-TIM3 (LY3321367) or anti-TIGIT (BMS-986207), are being currently tested, notably in breast cancer, and frequently with anti-PD(L)1 (e.g., NCT02913313 and NCT03600090) [116]. Similarly, activating antibody targeting co-stimulatory molecules like OX40 (PF-04518600, NCT03971409) or 4-1BB (urelumab, PF-05082566, NCT03364348) are also in development in breast cancer, notably in the AVIATOR study with avelumab (NCT03414658) [117].

Finally, another way to enhance the ICI responses is to combine them with intratumoral immunotherapy (e.g., with a TLR9 agonist, PAMPs or Ox40 Ab, [118,119]) or to use oncolytic viruses to increase tumor neo-antigens [120,121].

## 7. Conclusions

Immunotherapy with anti-PD-1/PD-L1 agents is emerging as a new treatment modality in breast cancer. But, compared to other cancers where several agents are already approved, we are at the beginning. Anti-PD-1/PD-L1 agents as monotherapy have demonstrated encouraging results in the metastatic setting, notably when administered earlier in the course of disease, although combination strategies seem to augment the responses. A few randomized studies with anti-PD-1/PD-L1 agents are completed or ongoing in combination with chemotherapy in the metastatic setting, as well as in the neo-adjuvant (and adjuvant) setting, where encouraging pCR rates have been obtained. Promising results have also been reported in combination with targeted therapies in the metastatic setting, and randomized trials are ongoing. On March 2019, the US Food and Drug Administration (FDA) granted an accelerated approval to atezolizumab in combination with nab-paclitaxel for adult patients with advanced TNBC whose tumors express PD-L1 as determined by an FDA-approved test.

There is no doubt that anti-PD-1/PD-L1 agents are going to complete the therapeutic armament of breast cancer in the near future. In this context, the identification of biomarkers able to predict for a clinical benefit of PD-1/PD-L1 inhibitors is fundamental to avoid very expensive and potentially toxic therapies for patients that are unlikely to benefit from them. Several predictors assessed in the tumor and/or its microenvironment are being developed (Teng,f cancer letters 2018). To date, PD-L1 expression measured using IHC is the only FDA-approved test. Like every IHC test, an important challenge for optimal use in clinical routine will be its standardization. The ongoing clinical trials will help to address this crucial issue.

## Figures and Tables

**Table 1:** Major published clinical trials using PD(L)-1 inhibitors in breast cancers.

Ph.	Anti-PD(L)-1	Single (S) or Combination	Study Title	Conditions or Disease	Treatment Line	Comparative Arm (for Phase IIR/III)	ORR (+/− 95% CI)	Duration of Response Median, Months (+/− 95% CI)	PFS Median, Months (+/− 95% CI)	OS Median, Months (+/− 95% CI)	Ref.
I	Atezolizumab	S	PCD4989g	M+ TNBC	≥1 L	/	1 L = 24% ≥2 L = 6%	21 mo	1.4 (1.3–1.6)	17.6 mo (10.2–NR)	Emens JAMA Oncol 2019
Ib	Pembrolizumab	S	KEYNOTE-012	M+ TNBC	≥1 L	/	18.5%	NA	6-mo PFS rate = 24%	12-mo OS rate = 43%	Nanda, BJC 2018
Ib	Pembrolizumab	S	KEYNOTE-028	HR+ HER2− PDL1+ LA or M+ BC	≥1 L	/	12.0% (2.5–31)	12.0 mo (7.4–15.9 mo).	NA	NA	Rugo, Clin Cancer Research 2018
Ib	Pembrolizumab	Chemotherapy (6 cohorts)	KEYNOTE-173	LA TNBC	Neo-adj	/	Overall pCR = 60% (30–85)	NA	NA	NA	Schmid, SABCS 2018
Ib	Pembrolizumab	Abemaciclib	JPCE	HR+, HER2− M+ BC	2 L/3 L	/	14.3%	NA	NA	NA	Rugo SABCS 2017 Tolaney, ASCO 2018
Ib	Avelumab	S	JAVELIN	M+ BC	≥1 L	/	Overall: 3% TNBC: 5.2%	NA	NA	NA	Breast Cancer Res Treat. 2018
Ib	Atezolizumab	Nab-paclitaxe	GP28328	M+ TNBC	1 L–3 L	/	39.4% (22–57)	NA	5.5 mo (5.1–7.7)	14.7 mo (10.1–NR)	Adams JAMA Oncol 2018
Ib/II	Pembrolizumab	Trastuzumab	KEYNOTE-014 (PANACEA)	Trastuzumab resistant		/	PDL1+ = 15% PDL1− =	3.5 mo (2.7–NR)	PDL1+:2.7 mo (2.6–4.0) PDL1−: 2.5 mo (1.4–2.7)	PDL1+: NR PDL1−: 7.0 mo (4.9–9.8)	Loi Lancet Oncol 2019
II	Durvalumab	Olaparib	MEDIOLA	HER2-negative gBRCAm M+ BC	≥1 L	/	63% (44–80%)	9.2 mo	8.2 mo	NA	Domchek, SABCS 2018
II	Pembrolizumab	S	KEYNOTE-086	M+ TNBC	≥2 L	/	5.3% (2.7–9.9)	NR	Median: 2.0 mo (1.9–2.0)	Median 9.0 mo (7.6–11.2)	Adams, Annals Oncol 2019
II	Pembrolizumab	Capecitabine	NCT03044730	LA or M+ hormone-refractory or TNBC	≥2 L	/	14%	NA	Median: 4.1 mo (2.3–8.2)	Median 15.4 mo (8.2–16.6 mo)	Shah ASCO 2019 (#1096)
II	Pembrolizumab	Paclitaxel or Capecitabine	NCT02734290	LA or M+ TNBC	1 L or 2 L	/	Cape = 43% Taxol = 25%	NA	NA	NA	Page ASCO 2019 (#1015)
II	Pembrolizumab	Niraparib	TOPACIO	LA or M+ TNBC	1 L to 5 L	/	21% (12–33)	NA	Median: 2.5 mo (2.3–8.2)	NA	Vinayak, JAMA Oncol 2019
II	Pembrolizumab	S	TAPUR	M+ BC, high TMB (≥ 9 Muts/Mb)	≥3 L	/	21% (8–41)	NA	Median: 2.6 mo	Median: 7.9 mo	Alva ASCO 2019 (#1014)
II	Atezolizumab	(Nab) paclitaxel + Cobimetinib	COLET	LA or M+ TNBC	1 L	/	34%	NA	6-mo PFS rate: 40.5%	6-mo OS rate: 84.1%	Brufski ASCO 2019 (#1013)
II-R	Pembrolizumab	Standard Chemo	I-SPY 2 trial	LA TNBC	Neo-adj	Placebo	Pembro: 62% Placebo: 22%	NA	NA	NA	Nanda ASCO 2017
II-R	Pembrolizumab	Eribulin	KEYNOTE-150 (ENHANCE 1) (Study 218)	M+ TNBC	1 L to 3 L	Eribulin +/− Pembrolizumab	26.4% (2017) Equal in 2 arms (2019)	8.3 mo (SABCS 2017)	P + E = 4.1 mo (ASCO 2019) E = 4.2 mo (ASCO 2019)	Median 17.7 (13.7–NR) (SABCS 2017)	Tolaney, SABCS 2017 Tolaney, ASCO 2019 (#1004)
II-R	Nivolumab	Doxo or Cyclo or RT (3*8 Gy)	TONIC	M+ TNBC	1 L to ≥3 L	Doxo or Cyclo or RT	Doxo = 35% Cyclo = 8% RT = 8%	NA	NA	NA	Voorwerk Nature Med 2019
II-R	Durvalumab	Nab-paclitaxel + standard EC	GeparNuevo	LA TNBC (cT2-cT4a-d)	Neo-adj	Placebo	pCR Durva: pCR placebo: 44%	NA	NA	NA	Loibl Annals Oncol 2019
III	Pembrolizumab	S	KEYNOTE-119	M+ TNBC	2 L or 3 L	single-agent CT (physician’s choice)	4.8%	NA	NA	not superior to CT	Merck press release
III	Atezolizumab	Nab-paclitaxel	IMPASSION-130	LA or M+ TNBC	1 L	Nab-paclitaxel	Atezo: 56% Placebo: 46%	HR= 0.78 (0.63–0.98) Median DOR Atezo: 7.4 mo Median DOR placebo: 5.6	HR 0.62 (0.49–0.78) Median PFS Atezo: 7.2 mo Median PFS placebo: 5.5 mo	HR 0.86 (0.72–1.02 Median OS Atezo: 21.0 mo Median OS placebo: 18.7 mo	Schmid NEJM 2018 Schmid ASCO 2019

Abbreviations: Ph = phase; IIR = phase II Randomized; TNBC: Triple Negative Breast Cancer; LA = Locally Advanced; M+ = metastatic; ORR = Objective Response Rate; DOR = Duration of Response; PFS = Progression-Free-Survival; OS = Overall Survival; L = Line; mo = months; NR = Not Reached; gBRCAm = germline BRCA-mutated;

## Supplementary Materials

The following are available online at https://www.mdpi.com/2072-6694/11/7/1033/s1, Table S1: Table of interventional studies with anti PD-1 or PDL1 agents recruiting.
