# Supplementary material for: PD-1/PD-L1 Targeting in Breast Cancer: The First Clinical Evidences are Emerging—A Literature Review
Source: Cancers (Basel). 2019 Jul 22;11(7):1033. doi: 10.3390/cancers11071033 (PMC6679223; doi:10.3390/cancers11071033)
Supplement: Supplementary file 1 [file cancers-11-01033-s001.pdf]

Supplementary Materials: PD-1/PD-L1 Targeting in Breast Cancer: the First Clinical Evidences are Emerging. A Literature Review

Table S1. Interventional studies with anti PD-1 or PDL-1 agents recruiting. Non-intraveinous administrations are indicated between brackets.

Ph.	Anti-PD(L)-1	Single (S) or Combination	Study title	NCT	Conditions or disease	Estimated Enrollement (n=)	Estimated Study Completion Date	Sponsor
1	Pembrolizumab	S	MK-3475 for Metastatic Inflammatory Breast Cancer (MIBC)	02411656	Inflammatory and metastatic breast cancer (IBC) or mTNBC	35	June 2020	MD Anderson
1	Ipilimumab Nivolumab	Entinostat [PO]	Entinostat, Nivolumab, and Ipilimumab in treating patients with solid tumors that are metastatic or cannot be removed by surgery or locally advanced or metastatic HER2-NegativeBreast Cancer	02453620	Breast carcinoma: HER2 negative, Invasive BC, Metastatic BC BC stage III, IIIA, IIIB, IIIC, IV Unresectable solid neoplasm	45	December 2019	NCI
1	Durvalumab	PVX-410 [IM]	Adjuvant PVX-410 Vaccine and Durvalumab in stage II/III Triple Negative Breast Cancer	02826434	TNBC stage II, III; HLA-A2 positive by deoxyribonucleic acid (DNA) sequence analysis	20	December 2022	Massachusetts General Hospit
1	Pembrolizumab	Nab-paclitaxel	A Study of changes in PD-L1 expression during preoperative treatment with Nab-Paclitaxel and pembrolizumab in Hormone Receptor (HR) Positive BC	02999477	HR positive breast cancer	50	January 2023	Dana-Farber
1	JS001 (anti PD1)	Cisplatine Gemcitabine	PD1 Antibody + GP as first line treatment for triple negative breast cancer (TNBC) Patients	03251313	TNBC	33	December 2020	Fudan University
1	PDR001 (anti PD1)	Ribociclib (LEE011) (cyclin D1 / CDK4-6 inhibitor) [PO] Fulvestrant [IM]	Ribociclib + PDR001 in BC and Ovarian Cancer	03294694	Metastatic HR+, HER2BC Metastatic Epithelial Ovarian Cancer	60	January 1 2022	Dana-Farber
1	Pembrolizumab	PVX-410 [vaccine]	PVX-410 Vaccine plus Pembrolizumab in HLA-A2+ metastatic TNBC	03362060	Metastatic Triple Negative BC	20	December 2024	Massachusetts General Hospit
1	Anti-PD-L1 / TGFbetaRII Fusion Protein M7824	Eribulin Mesylate	M7824 and eribulin mesylate in treating patients with metastatic TNBC	03579472	Anatomic or prognostic Stage IV BC HER2 -, RP -, RE – ; TNBC	20	October 31 2019	M.D. Anderson Cancer Center
1-2	Durvalumab	Paclitaxel	Study of safety and efficacy of durvalumab in combination with paclitaxel in metastatic TNBC patients	02628132	Metastatic TNBC	34	December 2019	King Faisal Hospit
1-2	Atezolizumab	Bevacizumab Enitinostat Exemestane Fulvestrant Ipatasertib Tamoxifen [PO]	A study of multiple immunotherapy-based treatment combinations in Hormone Receptor (HR) Positive Human Epidermal Growth Factor Receptor 2 (HER2)-Negative Breast Cancer (MORPHEUS)	03280563	Breast neoplasms HR+ HER2with progression during or following treatment with a cyclin-dependent kinase (CDK) 4/6 inhibitor in the firstor second-line setting, such as palbociclib, ribociclib, or abemaciclib.	111	October 2022	Hoffmann-La Roche
1-2	Nivolumab	Trastuzumab-deruxtecan	Trastuzumab Deruxtecan (DS-8201a) with Nivolumab in advanced breast and Urothelial cancer	03523572	BC, Urothelial Carcinoma	99	September 2020	Daiichi Sankyo
1-2	Pembrolizumab	Pegylated Liposomal Doxorubicin	Phase 1b Study of Pegylated Liposomal Doxorubicin and Pembrolizumab in Endocrine-resistant BC (KEYDOX)	03591276	HR+, HER2subtype, stage III non-operable, or stage IV disease, who have received at least two lines of hormonal therapy (one of which included aromatase inhibitors)	15	June 15 2020	Shaare Zedek Medical Center
1-2	Durvalumab	Paclitaxel Carboplatin	Paclitaxel + Carboplatin + Durvalumab with or without Oleclumab for previously untreated locally recurrent inoperable or metastatic TNBC (SYNERGY)	03616886	TNBC	171	December 2022	Jules Bordet Institute
1-2	SHR1210 (anti-PD1)	Nab-paclitaxel	FUSCC Refractory TNBC Umbrella (FUTURE)	03805399	TNBC	140	June 2022	Fudan University
2	Durvalumab	S (maintenance)	SAFIR02_Breast Efficacy of Genome Analysis as a Therapeutic Decision Tool for Patients with metastatic Breast Cancer (SAFIR02_Breast)	02299999	Metastatic breast cancer	1460	December 2022	UNICANCER

2	Pembrolizumab	S	Study of Pembrolizumab (MK-3475) monotherapy for metastatic Triple-Negative Breast Cancer (MK-3475-086/KEYNOTE-086)	02447003	mTNBC breast cancer	285	October 2019	MSD
2	Atezolizumab	Nab-paclitaxel	Triple-Negative First-Line Study: Neoadjuvant Trial of Nab-Paclitaxel and MPDL3280A, a PDL-1 Inhibitor in patients with triple negative breast cancer	02530489	TNBC, first line	37	February 2022	M.D. Anderson
2	Durvalumab	Tremelimumab	MEDI4736 and Tremelimumab in treating patients with metastatic HER2 Negative Breast Cancer	02536794	HER2 Negative<br>Recurrent breast carcinoma<br>Stage IV breast Cancer	30	June 2020	Northwestern University
2	Pembrolizumab	Anastrozole [PO]<br>Doxorubicin<br>Exemestane [PO]<br>Letrozole [PO]	Pembrolizumab and Doxorubicin Hydrochloride or Anti-Estrogen therapy in treating patients with triple-negative or HR + Metastatic Breast Cancer	02648477	HR positive or negative; HER2 negative ; stage IV BC, TNBC	56	September 2019	City of Hope Medical Center
2	Pembrolizumab	Hormonal Therapy	A Study of Anti PD1 (Pembrolizumab) + Hormonal Therapy in HR-positive localized IBC Patients with non-pCR to neoadjuvant chemotherapy	02971748	Inflammatory breast cancer	37	December 2021	M.D. Anderson Cancer Center
2	Pembrolizumab	Radiation: SBRT<br>ADV/HSV-tk (IT Replication-defective adenovirus)	SBRT and Oncolytic Virus Therapy before Pembrolizumab for metastatic TNBC and NSCLC (STOMP)	03004183	Metastatic Non-small Cell Lung cancer<br>Metastatic TNBC	57	November 2023	Houston Methodist Cancer Center
2	Pembrolizumab	Cyclophosphamide 50mg [PO]	Evaluation of Pembrolizumab in Lymphopenic Metastatic breast Cancer patients treated with metronomic Cyclophosphamide (CHEMOIMMUNE)	03139851	Metastatic breast cancer	36	February 2020	Centre Leon Berard
2	Avelumab	Fulvestrant [IM]<br>Palbociclib [PO]	Palbociclib after CDK and endocrine therapy (PACE)	03147287	Metastatic Breast Cancer	220	December 2024	Dana-Farber Cancer Institute
2	Atezolizumab	Pegylated liposomal doxorubicin<br>Cyclophosphamide	Atezolizumab combined with immunogenic chemotherapy in patients with metastatic Triple-negative Breast Cancer (ALICE)	03164993	TNBC	75	March 2024	Oslo University Hospit
2	Atezolizumab	Carboplatine	Carboplatin with or without atezolizumab in treating patients with stage IV Triple Negative Breast Cancer	03206203	TNBC stage IV breast cancer	185	February 2022	Vanderbilt-Ingram Cancer Center
2	Pembrolizumab	Nab-paclitaxel<br>Epirubicin<br>Cyclophosphamide	Neoadjuvant Pembrolizumab (Pbr)/Nab-Paclitaxel Followed by Pbr/Epirubicin/Cyclophosphamide in TNBC (NIB)	03289819	Breast cancer	50	December 2019	Institut fur Frauengesundheit
2	Nivolumab	Ipilimumab<br>Peg-liposomal doxorubicin<br>Cyclophosphamide	Phase IIb study evaluating immunogenic chemotherapy combined with Ipilimumab and Nivolumab in Breast Cancer (ICON)	03409198	Breast Cancer Hormone Receptor Positive; tumor metastatic breast cancer	75	January 2025	Oslo University Hospit
2	Avelumab	Utomilumab (4-1BB agonist)<br>Vinorelbine<br>Trastuzumab	The AVIATOR Study: Trastuzumab and Vinorelbine with avelumab or avelumab & utomilumab in advanced HER2+ breast cancer	03414658	HER2-positive Metastatic BC who have progressed on prior Trastuzumab and Pertuzumab	100	May 2025	Dana-Farber Cancer Institute
2	Nivolumab	Carboplatin	Carboplatin +/Nivolumab in Metastatic TNBC	03414684	Metastatic TNBC	132	June 2025	Dana-Farber Cancer Institute
2	Nivolumab	Capecitabine [PO]	OXEL: Immune Checkpoint or Capecitabine or Combination Therapy as Adjuvant Therapy for TNBC with residual disease	03487666	TNBC	45	December 2022	Georgetown University
2	Pembrolizumab	VRP-HER2 vaccination	A Study to evaluate concurrent VRP-HER2 Vaccination and Pembrolizumab for patients with Breast Cancer	03632941	HER2+ Breast Cancer	39	October 2021	Duke University
2	Nivolumab	Ipilimumab	NIMBUS: Nivolumab plus Ipilimumab in metastatic hypermutated HER2-negative breast cancer	03789110	hypermutated HER2 negative breast cancer	30	October 2022	Dana-Farber Cancer Institute
2	Nivolumab	Ipilimumab<br>Capecitabine	Evaluate the clinical benefit of a post-operative treatment associating radiotherapy + nivolumab + Ipilimumab *vs* Radiotherapy + Capecitabine for TNBC Patients with Residual Disease (BreastImmune03)	03818685	TNBC	98	March 2022	Centre Leon Berard
2	Pembrolizumab	Tamoxifen	Pembrolizumab And Tamoxifen among women with advanced Hormone Receptor Positive Breast Cancer and Esr1 Mutation	03879174	Advanced Hormone Receptor Positive breast cancer and Esr1 mutation	25	August 2022	Mediclinic Middle East
2	Atezolizumab	Docetaxel<br>Carboplatin<br>Trastuzumab [SC]<br>Pertuzumab<br>Epirubicin	Improving pre-operative systemic therapy for Human Epidermal Growth Factor Receptor 2 (HER2) amplified breast cancer (PREDIX II HER2)	03894007	Early-stage breast cancer, HER2+ breast cancer	190	December 2034	Renske Altena

		CyclophosphamideTrastuzumab emtansine						
2	Pembrolizumab	neoadjuvant (F)EC-paclitaxel	Study of Immunotherapy in Combination With Chemotherapy in HER2-negative Inflammatory Breast Cancer (PELICAN)	03515798	HER2-negative Inflammatory BC	81	April 2025	Institut Paoli-Calmettes
3	Pembrolizumab	Carboplatin Paclitaxel Doxorubicin Epirubicin Cyclophosphamide Placebo	Study of Pembrolizumab (MK-3475) Plus Chemotherapy vs Placebo Plus Chemotherapy as neoadjuvant therapy and Pembrolizumab vs Placebo as adjuvant therapy in participants with TNBC (MK-3475-522/KEYNOTE-522)	03036488	TNBC	1174	September 2025	MSD
3	Atezolizumab	Paclitaxel Dose-dense Doxo Dose-dense Epi Cyclophosphamide	A study comparing atezolizumab (Anti PD-L1 Antibody) in combination with adjuvant anthracycline/taxane-based chemotherapy vs chemotherapy alone in patients with operable TNBC (IMpassion030)	03498716	TNBC	2300	December 2024	Hoffmann-La Roche
3	Pembrolizumab	Paclitaxel Doxorubicin Epirubicin Cyclophosphamide	Study of pembrolizumab (MK-3475) vs Placebo in combination with neoadjuvant chemotherapy & adjuvant endocrine therapy in the treatment of early-stage ER-Positive, HER2-Negative (ER+/HER2-) Breast cancer (MK-3475-756/KEYNOTE-756)	03725059	High-risk early-stage ER+/HER2-breast cancer	1140	January 2031	MSD
3	JS001 (anti-PD1)	Nab-paclitaxel	A study of first-line JS001 and Nab-paclitaxel vs placebo and nab-Paclitaxel in participants with advanced recurrent or metastatic TNBC (KEYSTONE)	03777579	TNBC	375	July 2020	CSPC ZhongQi Pharmaceutical

Abbreviations: Ph = phase; IIR = phase II Randomized, TNBC: Triple Negative Breast Cancer; ORR = Objective Response Rate, DOR = Duration of Response; PFS = Progression-Free-Survival, OS = Overall Survival; L = Line, mo = months.
